# Comparison of Patient and Expert Perceptions of the Attainment of Research Milestones in Parkinson's Disease

**DOI:** 10.1002/mds.28319

**Published:** 2020-10-01

**Authors:** Patrick Bodilly Kane, Daniel M. Benjamin, Roger A. Barker, Anthony E. Lang, Todd Sherer, Jonathan Kimmelman

**Affiliations:** ^1^ Biomedical Ethics Unit, Studies of Translation, Ethics and Medicine (STREAM) Research Group McGill University Montreal Quebec Canada; ^2^ Information Sciences Institute University of Southern California Marina del Rey California USA; ^3^ John van Geest Centre for Brain Repair, Wellcome Trust/Medical Research Council (WT/MRC) Cambridge Stem Cell Institute, Department of Clinical Neuroscience University of Cambridge Cambridge United Kingdom; ^4^ Edmond J. Safra Program in Parkinson's Disease and the Morton and Gloria Shulman Movement Disorders Clinic Toronto Western Hospital Toronto Ontario Canada; ^5^ The Michael J. Fox Foundation for Parkinson's Research New York New York USA

**Keywords:** Parkinson's disease; prediction; forecasting

## Abstract

**Background:**

Commentators suggest that patients have unrealistic expectations about the pace of research advances and that such expectations interfere with patient decision‐making.

**Objective:**

The objective of this study was to compare expert expectations about the timing of research milestone attainment with those of patients who follow Parkinson's disease (PD) research.

**Methods:**

Patients with PD and experts were asked to provide forecasts about 11 milestones in PD research in an online survey. PD experts were identified from a Michael J. Fox Foundation database, highly ranked neurology centers in the United States and Canada, and corresponding authors of articles on PD in top medical journals. Patients with PD were recruited through the Michael J. Fox Foundation. We tested whether patient forecasts differed on average from expert forecasts. We also tested whether differences between patient forecasts and the average expert forecasts were associated with any demographic factors.

**Results:**

A total of 256 patients and 249 PD experts completed the survey. For 9 of the 11 milestones, patients' forecasts were on average higher than those of experts. Only exercise therapy met our 10% difference threshold for practical significance. Education was the only demographic that predicted patient deviations from expert forecasts on milestone forecasts. Patients offered significantly higher forecasts than experts that the clinical trials used in milestone queries would report positive primary outcomes.

**Conclusions:**

Differences between patient and expert expectations about research milestones were generally minor, suggesting that there is little cause for concern that patients who follow PD research are unduly swayed by inaccurate representations of research advancement in the media or elsewhere. © 2020 The Authors. *Movement Disorders* published by Wiley Periodicals LLC on behalf of International Parkinson and Movement Disorder Society

Because of the slow and inexorable progression of Parkinson's disease (PD), many patients with PD are keenly interested in emerging treatment strategies. However, the quality of information available to patients varies. Media coverage of emerging treatment approaches such as stem cell therapy^1^, ^2^, ^3^, ^4^, ^5^, ^6^ or repurposed drugs^7^ have often overstated the promise and understated the challenges for developing new treatments. Such representations may foster unrealistic expectations in patients^8^, ^9^ that can frustrate informed consent, clinical trial recruitment, or interfere with patient decision‐making.^10^, ^11^

Despite the very large literature on media representations of medical advances, little is known about how well patient perceptions of the promise and timelines for development of new treatments align with those of experts. Many studies suggest that patients who enroll in clinical trials harbor higher expectations of direct benefit than the physicians running the trials (“therapeutic overestimation”).^12^, ^13^ However, such studies cannot distinguish between different interpretations of probabilistic statements offered by patients.^14^, ^15^, ^16^ Nor does this body of literature offer a clear picture on patient perceptions of medical advance more generally.^17^

Medical and research communities have important responsibilities to communicate to patient communities their expectations about the promise and prospects of emerging treatments. Ideally, such communications should enable motivated patients to have a reasonable grasp of the maturity of new treatment paradigms so that patients can more effectively plan their current and future care.

We therefore undertook a study that solicited forecasts from patients with PD and experts about timelines for the attainment of 11 different PD research milestones. We then assessed the extent to which patient expectations aligned with those of experts.^18^

## 1 Methods

### 1.1 Expert and PD Patient Sample

Patients with PD were recruited to the survey through the Michael J. Fox Foundation for Parkinson's Disease Research (MJFF) e‐mail list. To minimize survey burden, each e‐mail address was solicited by email once for participation. Because the MJFF e‐mail list contains both patients with PD and nonpatients, our survey responses contained both patient and nonpatient responses. For the purposes of this study, we removed the nonpatient responses from our sample to focus solely on patient perceptions. We report an analysis with all of the responses per our preregistration in Supplemental Material S1.

PD experts were recruited in the following 3 ways: (1) the MJFF database of experts, (2) the identifcation of doctors specializing in PD at the 25 neurology departments in the United States receiving top rankings in *US News and World Report* 2018 and 3 highly regarded hospital systems in Canada, and (3) the identifcation of corresponding authors of articles on PD from the past 5 years in 10 top general and neurology/movement disorder journals. Experts were solicited by email 3 times.

### 1.2 Survey

Milestones in PD research were generated by R.B., A.L., and T.S., seeking milestones whose attainment would be objectively verifiable, diverse, and thought to be of interest to the patient community (see Kane and colleagues^18^ for details). For milestones dealing with trial launches or enrollments, we asked for a prediction of whether the trial would be positive on a primary outcome. The survey was administered via Qualtrics. Expert versions of the milestones and survey wording are provided in Table 1.

**TABLE 1:** List of milestones used in our survey

Topic	Event	Full Description	Additional Patient Text
Monogenic gene therapy	US FDA approval	The US FDA approves a gene therapy directed at a monogenic cause of PD such as *LRRK2*, *GBA*, or parkin for treatment of PD.	Gene therapy is a technique that modifies a person's genes to treat or cure disease.
Precision medicine therapy	Trial enrollment; results	A rigorous phase 2 or phase 3 clinical trial PD that specifies eligibility based on *GBA* mutational status successfully enrolls at least 80 subjects.	*GBA* is the most commonly mutated gene in people with PD. The *GBA* mutation appears in between 5% and 10% of patients with PD.
Cell therapy	Trial initiation; results	The launch of a rigorous phase 2 or phase 3 clinical trial involving implantation of patients with PD with dopaminergic cells derived from pluripotent stem cells.	PD causes loss of dopamine producing cells in the brain (dopaminergic cells). Scientists believe that new dopaminergic cells grown from stem cells could 1 day restore normal dopamine levels in the brains of patients with PD.
Imaging	Trial initiation; results	A selective α‐synuclein imaging agent is integrated into a rigorous PD interventional clinical trial.	α‐synuclein is a protein believed to play a key role in PD progression. Scientists are developing an “imaging agent” to measure α‐synuclein in the brain, which could be used when testing PD treatments.
Deep brain stimulation	US FDA approval	US DA approval of the first closed‐loop deep brain stimulation device for the management of PD.	Scientists are trying to develop “smart” deep brain stimulation devices that can modulate their stimulation based on a patient's symptoms. These are called “closed loop” devices.
Treatment for PD‐MCI	Trial initiation; results	Launch of a rigorous phase 3 clinical trial testing a novel, noncholinesterase inhibiting drug in the treatment of PD‐MCI.	MCI—changes in memory or cognition that do not interfere with daily life—is common in PD. It is usually treated with a class of drugs called cholinesterase inhibitors. Scientists are trying to discover better drugs for treating MCI.
Drug repositioning	Trial results	A rigorous phase 3 clinical trial using a repositioned medication and aimed at slowing the progression of PD symptoms reports a positive outcome on a primary efficacy end point.	Sometimes drugs known to work for certain diseases (eg, cancer) are discovered to work against other diseases (eg, multiple sclerosis). When a drug is used to treat multiple diseases this way it is referred to as a “repurposed” drug.
Exercise therapy	Trial results	A rigorous phase 2 or phase 3 clinical trial testing the effect of exercises, physical activity, or physical therapy on PD progression reports a positive outcome on a primary efficacy end point.	None
Body worn sensors	Clinical practice guideline recommendation	An algorithm derived from a body worn sensor is accepted by the International Parkinson and Movement Disorder Society as a valid measure of PD symptoms.	Scientists are testing body worn sensors for measuring symptoms of PD.
Basic science discovery	Awarded *Science* magazine's Breakthrough of the Year	*Science* magazine awards “Breakthrough of the Year” to a molecule, process, cell, or discovery that is expressly described, in the accompanying *Science* article, as implicated in PD pathogenesis or possible treatment.	None
Immunotherapy	Trial results	A rigorous phase 2 or phase 3 clinical trial testing an alpha‐synuclein based immunotherapy for PD reports a positive outcome on a primary efficacy end point.	α‐synuclein is a protein believed to play a large role in PD progression. Scientists are trying to develop an “immunotherapy” that allows the immune system to target α‐synuclein and block its harmful effects.

Additional clarifying details for some of the milestones were listed as footnotes (see Supplemental Material S1 for details). US FDA, US Food and Drug Administration; PD, Parkinson's disease; MCI, mild cognitive impairment.

The patient survey was created in collaboration with the MJFF research communications directors by replacing jargon terms from the expert survey with more accessible language and providing background information on the context of why each milestone was important.

Our survey sought forecasts for milestone attainment in 4 time bins (next 2 years, 4–6 years, 6–10 years, and longer than 10 years). We expected the richness of this elicitation, in particular asking about all possible future time periods in which a milestone could occur, to limit the effects of various biases and ambiguities associated with simpler survey approaches. For the questions about the outcomes of clinical trials we used a simpler elicitation format where participants just indicated the probability of a positive outcome.

A preliminary run of the survey was conducted in October 2018 with 50 experts and 50 patients to test our distribution platforms. The full surveys were run in January 2019 (patients) and between January and April 2019 (experts).

### 1.3 Preprocessing of Forecasts

This survey was designed primarily to test the effect of aggregation techniques on forecast accuracy in the context of PD, the results of which will be reported in coming years. The present study was planned as a secondary objective. Adjusting forecasts obtained for the primary objective to enable analyses for this secondary objective required preprocessing for the present analysis (eg, converting 4 time bins into 2: the probability of each milestone occurring or not occurring in the next 6 years). Details of the preprocessing are reported in Supplemental Material S1.

### 1.4 Analysis

Our primary analysis was a 2‐sided bootstrapped *t* test comparing the average expert forecast of the milestone occurring in the next 6 years to the average patient forecast of the same for each milestone. We applied a Bonferroni correction to the individual milestone tests. After we had analyzed the expert data, but before analyzing the patient data, we defined a difference of 10% between patient and expert average forecasts as representing a meaningful difference in expectation. This threshold was based on our assumption that differences smaller than 10% would have little practical impact on patient decision‐making given the presence of other influences. We also applied this analysis to the additional questions about trial results.

As an exploratory analysis, we fitted a regression model to test whether any demographic variables predicted higher forecasts. For each forecast, we first subtracted the average expert forecast for the associated milestone. We then regressed this difference on age, gender, and education along with milestone controls. Education was coded as a factor with levels: high school, bachelor's, master's, doctor of medicine, doctor of medicine/doctor of philosophy, and doctor of philosophy. The questions about trial results were not included in this analysis.

Our survey received approval by the McGill institutional review board; participants provided consent online. This analysis was preregistered after the survey, and some preliminary analysis of the expert forecasts was completed (but before any analysis of the patient forecasts) and can be found on the Open Science Framework at https://osf.io/3zcws/?view_only=b008d83777e24c16a7768ffbde92ab0a. Our primary analysis was modified following suggestions by *Movement Disorders* peer reviewers (our unmodified analysis can be found in Supplemental Material S1; the changes made do not alter the substantive pattern of conclusions).

## 2 Results

### 2.1 Properties of Forecaster Samples

The survey was completed by 256 patients with PD of 13,896 lay people solicited (response rate is impossible to estimate because we do not know what proportion of the invitations were received). The survey was also completed by 63 lay people who were not patients with PD. The patient sample was 43% female; median age of respondents was 68 years (range 38–90); the highest degrees held by our respondents were high school (18%), bachelor's (36%), master's (29%), doctor of medicine (3%), doctor of medicine/doctor of philosophy (2%), doctor of philosophy (10%). The survey was completed by 249 experts in PD as previously described.^18^

### 2.2 Properties of Forecasts

**FIG. 1:**
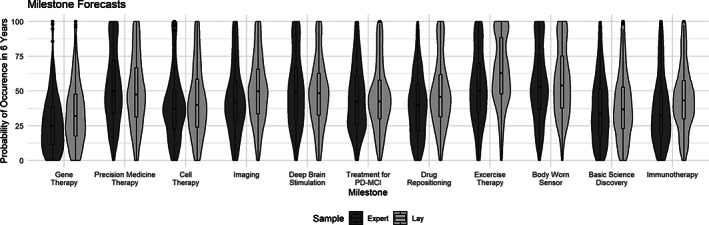
Violin plots of the dichotomized lay and expert forecasts for each milestone. PD‐MCI, Parkinson's disease–mild cognitive impairment.

Figure 1 displays the forecasts for each milestone for patient and expert participants. Table 2 contains the means and standard deviations of forecasts for each milestone for both the patient and expert participants as well as *P* values for bootstrapped *t* tests. On average, patients and experts both tended to offer higher forecasts for milestones related to disease management and diagnostics (eg, body worn sensors) than more experimental therapies (eg, gene therapy, stem cell therapy). In accordance with experts, patients rated the monogenic disease gene therapy milestone as the least likely to occur, but in contrast with experts, they rated the exercise therapy milestone as the most likely to occur. The variance of forecasts was similar for both groups with the distribution of forecasts covering almost the entire range of possible values for each milestone, indicating a diversity of expectations among both experts and patients. Overall, the distributions appear similar, although patients provided generally higher forecast probabilities, with the patient average being higher than the expert average for 9 of the 11 milestones.

**TABLE 2:** Means and SDs for probability of the milestone occurring in the next 6 years for each milestone for patients and experts

Milestone	Lay Mean (SD)	Expert Mean (SD)	*P* Value
Monogenic gene therapy	33.85 (22.49)	27.26 (20.39)	<0.001*
Precision medicine therapy	49.38 (25.50)	52.83 (26.73)	0.13
Cell therapy	42.64 (25.37)	40.00 (24.52)	0.23
Imaging	50.52 (23.98)	44.00 (24.74)	0.002*
Deep brain stimulation	48.39 (24.39)	50.77 (26.16)	0.29
Treatment for PD‐MCI	45.79 (23.55)	45.06 (23.85)	0.73
Drug repositioning	48.73 (24.48)	39.60 (23.24)	<0.001*
Exercise therapy	63.65 (25.27)	52.77 (25.63)	<0.001*
Body worn sensors	56.75 (25.40)	55.34 (26.51)	0.54
Basic science discovery	39.82 (24.55)	37.28 (24.24)	0.24
Immunotherapy	44.62 (23.16)	36.94 (24.75)	<0.001*
Precision medicine therapy result	56.22 (27.63)	44.73 (24.90)	<0.001*
Cell therapy result	58.71 (29.07)	39.73 (26.87)	<0.001*
Imaging result	62.79 (21.17)	49.53 (26.41)	<0.001*
Treatment for PD‐MCI result	59.56 (27.17)	39.90 (24.08)	<0.001*

The last 4 rows represent the questions about trial results. Kendall's τ for the patient and expert averages was 0.64, indicating moderate agreement on the rank order of milestones.

SD, standard deviation; PD, Parkinson's disease; MCI, mild cognitive impairment.

For our primary analysis, we observed a statistically significant difference in forecasts for several milestones (Table 2). However, only the exercise therapy milestone met our predefined level of practical significance of 10%. All of the additional questions about trial results showed statistically significant differences in expectations and met our benchmark for practical significance.

### 2.3 Analysis of Relationship Between Higher Lay Forecasts and Demographic Information

In our exploratory analysis, neither age (*t* = −1.20, *P* = 0.23) nor gender (*t* = −1.73, *P* = 0.08) provided significant increases in the fitting of the data. Only education provided a significant increase in fit (*F*_2724,5_ = 9.98, *P* < 0.001), with patients having only a bachelor's degree offering the highest forecasts of milestone attainment followed by those with only a high school diploma, and those holding postgraduate degrees offering the lowest forecasts. Estimates of the coefficients and 95% confidence intervals are presented in Table 3.

**TABLE 3:** Coefficient estimates and 95% confidence intervals for our regression of relative patient forecasts and their deviation from average expert forecasts by lay person age, gender, education, and PD status, along with milestone controls

Variable	Coefficient Estimate	95% Confidence Interval
Age	−0.07	−0.18 to 0.04
Gender (male)	−1.65	−3.51 to 0.20
Education (bachelor's)	3.87	1.35 to 6.40
Education (master's)	−2.83	−5.48 to −0.15
Education (MD)	−5.79	−11.3 to −0.22
Education (MD/PhD)	−4.28	−10.96 to 2.33
Education (PhD)	−4.20	−7.67 to −0.64

The estimates for the milestone controls are omitted.

PD, Parkinson's disease; MD, doctor of medicine; PhD, doctor of philosophy.

## 3 Discussion

Patients' forecasts about the attainment of major PD research milestones within 6 years were significantly and consistently higher than those of experts. However, misalignment was modest in magnitude. For only 1 milestone—exercise therapy—differences met our prespecified criterion of practical significance. Exceptionally high forecasts for exercise therapy may reflect the fact that many patients with PD have direct and positive experience with exercise, including that it is readily available and lacks major adverse effects.

Our finding that patients with graduate degrees tended to provide forecasts that were more in line with experts is consistent with previous work showing a relationship between low educational attainment and phenomena such as therapeutic misestimation.^13^, ^19^ We also observed significant variation in the judgments of both patient and experts, with many patients offering low probabilities and many experts offering high probabilities. This could suggest that patients have views that align more or less with expert opinion. However, it also raises the possibility that patients may be unduly influenced by physicians with extreme views.

Our core findings might be explained in several ways. First, if one accepts the premise that experts on average maintain an accurate read of the future, patients may harbor mildly unrealistic positive expectations about milestone attainment. Second, some patient expectations we captured may represent an expression of a hopeful attitude rather than purely probabilistic estimates.^14^, ^15^, ^16^ Third, higher patient forecasts could signal greater patient uncertainty about the timing of milestone attainment. We note that for many milestones, patient forecasts were closer to 50% than expert forecasts.

In contrast to the milestone forecasts, patients offered significantly and meaningfully higher forecasts about positive trial outcomes than experts. This may reflect a lack of experience with how often clinical trials report null results. Such high probabilities would be consistent with patients tending to overestimate the probability of benefit associated with trial participation.^12^, ^13^ Higher trial outcome forecasts could also be an artifact of the simpler format by which the trial result forecasts were obtained.

Our study has several limitations. We focused on specific milestones to ensure that these predictions were verifiable for a future study assessing accuracy. This emphasis on verifiability meant that our questions excluded other important research milestones. Moreover, we selected milestones that, at the outset, had some prospects of materializing within 10 years. The use of milestones that are widely regarded by experts as highly unlikely to occur within 10 years, such as the reversal of many PD disease features via stem cells, might have led to larger differences in expectations. Second, forecasts are sensitive to elicitation platforms. Although our preprocessing procedure likely blunted some effects of innumeracy, our question format may have interacted with opinions. Last, selection bias is always a concern in surveys of this nature. Our patient sample was enriched for highly informed and engaged patients. Indeed, participants were identified not from clinics but, rather, because they subscribe to electronic alerts from a major research foundation. Our sample was more educated generally and more informed about PD specifically than would be a random sample of patients with PD.^20^

Our study investigated attitudes about broad lines of research rather than beliefs about specific clinical trials. It thus marks a contrast with previous work on patient expectations in clinical research.^12^, ^13^ The fact that patient forecasts did not exceed those of experts by large amounts across a wide frontier of innovation is inconsistent with a common perception that patients uncritically absorb exuberant media reports or commercial claims. Indeed, our findings of alignment are consistent with research suggesting that even patients with PD seeking out unproven interventions tend to harbor very modest and skeptical expectations about benefits for unproven treatments.^21^, ^22^ Nevertheless, we did observe greater misalignment for patients with lower education attainment compared with those with graduate degrees. We also observed higher expectations about trials producing positive results on their primary end points. This last finding, although a secondary outcome of our study and in need of replication, is potentially concerning because it may bear on patient consent to trial enrollment. Many promising PD treatments have produced null results when tested in randomized trials, and recent meta‐analyses suggest that there is no medical advantage to being assigned to treatment arms in placebo‐controlled trials involving neurodegenerative diseases.^23^ Our findings suggest that many patients may not appreciate that randomized trials in PD begin with a credible and honest null hypothesis. Physicians, professional societies, and patient advocacy organizations might better explain to patients and patient communities that even PD treatments backed by sound preclinical and early‐phase evidence have a low prior probability of demonstrating efficacy in randomized trials.

## Author Roles

(1) Research Project: A. Conception, B. Organization, C. Execution; (2) Statistical Analysis: A. Design, B. Execution, C. Review and Critique; (3) Manuscript: A. Writing of the First Draft, B. Review and Critique.

P.B.K.: 1C, 2A, 2B, 2C, 3A

D.M.B.: 1A, 1B, 1C, 2A, 2C, 3B

R.A.B.: 1A, 3B

A.E.L.: 1A, 3B

T.S.: 1A, 3B

J.K.: 1A, 1B, 1C, 2C, 3B

## Full financial disclosures for the previous 12 months

J.K. serves on Data Safety Monitoring Boards (DSMBs) for Ultragenyx and National Institute for Allergy and Infectious Disease (NIAID) for which he received compensation. R.A.B. receives royalties from Springer and Wiley. He provides consultancy services to Living Cell Technologies, Fujifilm Cellular Dynamics Inc, BlueRock Therapeutics, Sana Biotherapeutics, Novo Nordisk, and UCB. A.E.L. provides consultancies to Abbvie, Acorda, Biogen, Bristol Myers Squibb, Intracellular, Janssen, Jazz, Lilly, Lundbeck, Merck, Ono, Paladin, Roche, Seelos, Syneos, Sun Pharma, Theravance, and Corticobasal Degeneration Solutions; serves on the advisory boards of Jazz Pharma, PhotoPharmics, and Sunovion; and has received honoraria from Sun Pharma, AbbVie, and Sunovion. P.B.K., D.M.B., and T.S. have nothing to report.

## Supporting information


**Appendix** **S1** Supporting Information.
