# Supplementary material for: Comparison of Patient and Expert Perceptions of the Attainment of Research Milestones in Parkinson's Disease
Source: Mov Disord. 2020 Oct 1;36(1):171–7. doi: 10.1002/mds.28319 (PMC7891331; doi:10.1002/mds.28319)
Supplement: Supplementary file 1 — Appendix S1 Supporting Information. [file MDS-36-171-s001.docx]

**Expert Recruitment**

We performed a corresponding author search on the following journals: *BMJ*, *Lancet*, *PLoS Med*, *NEJM*, *JAMA*, *JAMA Neurology*, *Lancet Neurology*, *Neurology*, *Annals of Neurology* and *Movement Disorders*.

**Milestone Items**

The full milestone text including footnotes for the patient and expert surveys are found in tables 1A and 2A.

**Methods**

This survey was designed for the purposes of testing the effect of aggregation techniques on forecast accuracy in the context of PD. The present study was planned as a secondary objective. Specifically, the survey was designed to test an aggregation technique that weighs forecasts based on the presence of violations of the laws of probability (we refer to these violations as incoherence and their absence as coherence).^1,2^ The basic idea behind this approach is that people who violate the laws of probability did so because they had a less well thought out answer to the question, and thus that answer should receive less weight in aggregation.

In order to perform coherence weighting the elicitation must allow participants to violate the laws of probability. In our case we did this by asking participants to make predictions about the probability of a milestone occurring in one of four mutually exclusive time bins (within the next 2 years, within the next 4-6 years, within the next 6-10 years and not in the next 10 years). Because these time bins were mutually exclusive and covered the complete range of possibilities the probability participants assigned across them should add up to 100. However, we did not force participants to provide probabilities that added up in this way. Additionally, to make it more difficult for participants to trivially provide coherent probabilities we asked one of the time bins (not in the next 10 years) separately from the others. Thus participants had to both anticipate that there was at least one more time bin comprising the remaining possibilities and rely on what they remembered about their previous predictions to give a coherent response.

One of the challenges of this approach is that highly incoherent predictions are essentially worthless. Rather than drop them from the sample entirely, after elicitation we performed a procedure on them to make them into coherent forecasts. This procedure consisted of finding the closest set of coherent forecasts in terms of Euclidean distance and replacing the incoherent forecasts with coherent forecasts. This procedure amounts to solving the constrained optimization problem,

$$\min_{p^{'}} \left( p_{<2}-{p^{'}}_{<2} \right)^{2}+\left( p_{4-6}-{p^{'}}_{4-6} \right)^{2}+\left( p_{6-10}-{p^{'}}_{6-10} \right)^{2}+\left( p_{>10}-{p^{'}}_{>10} \right)^{2}$$

$$s.t. {p'}_{<2}+{p'}_{4-6}+{p^{'}}_{6-10}+{p^{'}}_{>10}=1 and p^{'}\geq0$$

Where the $p$ are the elicited probabilities and the $p'$ are the “coherentized” probabilities. This procedure was carried out for each participant for each milestone. As an example if a participant provided the probabilities 30%, 40%, 40%, 30% for the four time bins respectively the total would be 140% and thus their response would be incoherent. The nearest set of coherent probabilities is given by 20%, 30%, 30%, 20% thus these would be the coherentized responses for that milestone for that participant. Throughout the paper and in the supplemental materials we exclusively report the coherentized probabilities.

Additionally, to facilitate analysis we dichotomized each forecast by combining the forecasts for the within the next 2 years and within the next 4-6 years bins, reducing the four bins to a single judgment of the probability the milestone will occur in the next 6 years or not. The choice of where to dichotomize was essentially arbitrary, and thus we present the results of our primary analysis for two other dichotomizations: a within the next 2 years versus not within the next 2 years dichotomization and a within the next 10 years versus not within the next 10 years dichotomization.

Finally, the analysis we report in the paper represents an alteration of our originally preregistered analysis based on the comments made during the review process. Our original analyses compared the average lay predictions to the aggregated expert predictions previously reported.^3^ These aggregated expert predictions were the result of performing coherence weight on the expert predictions. This analysis also includes all of the response to the patient survey, not just those of actual PD patients. We report the results of this pre-registered analysis here.

**Results**

The results of our primary analysis for the alternate dichotomizations are found in Tables 3A and 4A for the within the next 2 year and within the next 10 year dichotomizations. The overall pattern of results is very similar between these two alternate ways of dichotomizing the predictions and our original analysis. While the pattern of statistical significance was largely the same, here were two differences in the pattern of significance. For the within the next 2 year dichotomization, the difference between lay and expert expectations for the body worn sensors milestone reached statistical significance. For the within 10 year dichotomization the difference between lay expert expectations for the imagine milestone did not reach significance. In terms of practical significance only the exercise therapy milestone reached the predefined difference of 10% for the within 2 year dichotomization, while the monogenic gene therapy milestone was the only one to reach the predefined threshold for the 10 year dichotomization.

The results for our preregistered analysis can be found in Table 5A. The pattern of the results is exactly the same as for the primary analysis reported in the paper, with the same milestones achieving statistical and practical significance.

| **Topic** | **Event** | **Full description** | **Footnotes** |
| --- | --- | --- | --- |
| Monogenic Gene Therapy | FDA approval | Gene therapy is a technique that modifies a person’s genes to treat or cure disease.  **The FDA approves* a gene therapy**† for the treatment of Parkinson’s disease.** | *Approvals must be announced at accessdata.fda.gov with an approval date on or before the specified date.  **The gene therapy must be non-alpha synuclein Parkinson-related, and use a viral vector or liposome to deliver the therapy (i.e. directed at a gene that can cause PD such as LRRK2, GBA, parkin, etc.)  † Gene therapies can work by several mechanisms: 1) Replacing a disease-causing gene with a healthy copy of the gene; 2) Inactivating a disease-causing gene that is not functioning properly; 3) Introducing a new or modified gene into the body to help treat a disease (from: https://www.fda.gov/BiologicsBloodVaccines/CellularGeneTherapyProducts/ucm573960.htm) |
| Precision Medicine Therapy | Trial Enrolment; results | GBA is the most commonly mutated gene in people with Parkinson's. The GBA mutation appears in between 5 and 10 percent of patients with Parkinson’s disease.  **A rigorous clinical trial* testing a treatment designed for Parkinson's patients with GBA mutations** successfully enrolls at least 80 subjects.***** | *Registered on clinicaltrials.go with a “first posted” date on or before the date specified. Trial must be phase 2 (a small study that provides a preliminary picture of efficacy) or phase 3 (a large study that formally tests efficacy), must be a randomized controlled trial with a sham/placebo arm, and must have long-term follow up of at least 2 years using both clinical and imaging measures.  **GBA stands for Glucocerebrosidase; patients with Parkinson’s disease and associated Lewy body disorders have an increased frequency of GBA mutations (deviations from the norm) compared to individuals without Parkinson’s. (Sidransky E, et al. The link between the GBA gene and parkinsonism. Lancet Neurol. 2012;11(11):986-998.)  ***As determined by a press release from the sponsor, a final enrollment record on clinicaltrials.gov, a published abstract or article available on PubMed, or direct confirmation from contacts listed on clinicaltrials.gov. |
| Cell Therapy | Trial initiation; results | Parkinson's disease causes loss of dopamine producing cells in the brain (dopaminergic cells). Scientists believe that new dopaminergic cells grown from stem cells could one day restore normal dopamine levels in the brains of Parkinson’s disease patients.  **The launch* of a rigorous clinical trial** testing dopaminergic cell therapy*** in Parkinson’s patients.** | *‘Launch’ means a trial is registered on clinicaltrials.gov with a “first posted” date on or before the date specified.  **The trial must be either must be either phase 2 (a small study that provides a preliminary picture of efficacy) or phase 3 (a large study that formally tests efficacy), and must be a randomized controlled trial with a sham/placebo arm, must have long-term follow up of at least 2 years using both clinical and imaging measures, and must plan to enroll at least 120 patients.  ***By “dopaminergic cell therapy” we mean a dopamine cell that has been made from a human embryonic stem cell or induced pluripotent stem cell source |
| Imaging | Trial initiation; results | Alpha-synuclein is a protein believed to play a key role in Parkinson’s disease progression. Scientists are developing an "imaging agent" to measure alpha-synuclein in the brain, which could be used when testing Parkinson's treatments.  **A selective alpha synuclein imaging agent* is used in a trial testing a treatment for Parkinson's disease.**†** | *PET (positron emission topography) radiotracer to image the distribution of alpha-synuclein within the brain (https://www.michaeljfox.org/foundation/grant-detail.php?grant_id=845)  **The trial must be a randomized controlled trial with a sham/placebo arm, must have long-term follow up of at least 2 years using both clinical and imaging measures, must enroll at least 120 patients if phase 2, and must be registered on clinicaltrials.gov with a “first posted” date on or before the date specified.  †A clinical study in which participants are assigned to receive one or more interventions (or no intervention) so that researchers can evaluate the effects of the interventions on biomedical or health-related outcomes (from: https://clinicaltrials.gov/ct2/about-studies/glossary). |
| Deep Brain Stimulation | FDA approval | Scientists are trying to develop “smart” deep brain stimulation devices that can modulate their stimulation based on a patient’s symptoms. These are called “closed loop” devices.  **FDA approval* of the first closed-loop deep brain stimulation** device for the treatment of Parkinson’s disease.** | *Approvals must be announced at accessdata.fda.gov with an approval date on or before the specified date.  **Open-loop devices stimulate at constant intensity regardless of current symptoms, while development of a closed-loop device will allow for an implanted neurostimulator that will use sensors and real-time modulation to determine the level of stimulation required based on patient symptoms at that moment in time (http://brl.ee.washington.edu/neural-engineering/closed-loop-dbs/). |
| Treatment for PD-MCI | Trial initiation; results | Mild cognitive impairment- changes in memory or cognition that do not interfere with daily life- is common in Parkinson’s disease. It is usually treated with a class of drugs called cholinesterase inhibitors. Scientists are trying to discover better drugs for treating mild cognitive impairment.  **Launch* of a rigorous clinical trial** testing a novel, non-cholinesterase inhibiting drug*** in the treatment of Parkinson's disease mild cognitive impairment.****** | *Launch means a trial of the specified phase is registered on clinicaltrials.gov with a “first posted” date on or before the date specified.  ** The trial must be phase 3 (a large study that formally tests efficacy), must be a randomized and controlled trial with a sham/placebo arm, must have long-term follow up of at least 2 years using clinical measures, and must enroll at least 250 patients.  ***By “novel” we mean drugs that do not work the same way as currently approved drugs for treating this disorder - “cholinesterase inhibition.”  ****Mild cognitive impairment in Parkinson’s disease (PD-MCI) diagnostic criteria are defined based on the Movement Disorder Society (Litvan I, et al. Diagnostic Criteria for Mild Cognitive Impairment in Parkinson’s Disease: Movement Disorder Society Task Force Guidelines. Movement Disorders. 2012; 27(3):349-356). |
| Drug re-positioning | Trial results | Sometimes, drugs known to work for certain diseases (e.g. cancer) are discovered to work against other diseases (e.g. multiple sclerosis). When a drug is used to treat multiple diseases this way it is referred to as a "repurposed" drug.  **A rigorous clinical trial* will show that a repurposed drug** can be used to significantly*** slow the progression of symptoms of Parkinson’s disease.****** | *The trial must be phase 3 (a large study that formally tests efficacy), must be a randomized controlled trial with a sham/placebo arm, must have long-term follow up of at least 2 years using both clinical and imaging measures, and must enroll at least 250 patients.  **A drug developed and FDA approved for one disorder and used to treat or manage a completely different disorder.  ***As defined by statistical significance on all prespecified primary endpoint(s) that were prospectively registered on clinicaltrials.gov; at least one of the primary endpoints must use a common Parkinson's disease efficacy measure.  ****Results must be accessible on PubMed or clinicaltrials.gov with a date on or before the date specified. |
| Exercise therapy | Trial results | **A rigorous clinical trial* testing the effect of exercises, physical activity, or physical therapy on Parkinson’s progression reports** positive results.***** | * The trial must be either must be either phase 2 (a small study that provides a preliminary picture of efficacy) or phase 3 (a large study that formally tests efficacy), and must be a randomized controlled trial with a sham/placebo arm, must have long-term follow up of at least 2 years using both clinical and imaging measures, and must enroll at least 120 patients.  **Results must be accessible on PubMed or clinicaltrials.gov.  ***As determined by a press release from the sponsor, a final enrollment record on clinicaltrials.gov, a published abstract, or direct confirmation from contacts listed on clinicaltrials.gov; at least one of the primary endpoints must use a common Parkinson's disease efficacy measure. |
| Body worn sensors | Clinical practice guideline recommendation | Scientists are testing body worn sensors for measuring symptoms of Parkinson’s disease.  **The most important organization of physicians who treat Parkinson’s Disease, the International Parkinson and Movement Disorder Society, endorses* the use of a body worn sensor technique** for measuring symptoms of Parkinson’s disease.** | *Announced as an acceptable measure at either an International Parkinson and Movement Disorder Society conference, in the International Parkinson and Movement Disorder Society journal Movement Disorders, or in news and updates on [www.movementdisorders.org](http://www.movementdisorders.org).  **Body-worn sensors such as wrist sensors, shoe insoles, and smartphone apps allow for the remote monitoring of Parkinsonian motor symptoms to assist in treatment decisions and evaluation of treatment plans  (Fisher J, et al. (2016) Body-Worn Sensors in Parkinson’s Disease: Evaluating Their Acceptability to Patients. Telemed J E Health. 22(1):63-69.) |
| Basic science discovery | Awarded *Science* magazine’s Breakthrough of the Year | ***Science* magazine awards "Breakthrough of the Year"* to a discovery or event that is expressly described, in the magazine article, as implicated the development of or possible treatment for Parkinson's disease***.* | *For more information on Breakthrough of the Year: http://vis.sciencemag.org/breakthrough2017/. |
| Immuno-therapy | Trial results | Alpha-synuclein is a protein believed to play a large role in Parkinson’s disease progression. Scientists are trying to develop an "immunotherapy" that allows the immune system to target alpha-synuclein and block its harmful effects.  **A rigorous clinical trial* testing an alpha-synuclein based immunotherapy** for Parkinson’s disease reports*** positive results.****** | *The trial must be either must be either phase 2 (a small study that provides a preliminary picture of efficacy) or phase 3 (a large study that formally tests efficacy), and must be a randomized controlled trial with a sham/placebo arm, must have long-term follow up of at least 2 years using both clinical and imaging measures, and must enroll at least 120 patients.  **Immunotherapy could be active or passive.  ***Results must be accessible on PubMed or clinicaltrials.gov with a date on or before the date specified.  ****As defined by statistical significance on all prespecified primary endpoint(s) that were prospectively registered on clinicaltrials.gov; at least one of the primary endpoints must use a common Parkinson's disease efficacy measure. |

**Table 1A**: List of milestones used in the patient survey including footnotes. The text of the milestone itself was presented in bold.

| **Topic** | **Event** | **Full description** | **Footnotes** |
| --- | --- | --- | --- |
| Monogenic Gene Therapy | FDA approval | The FDA approves* a gene therapy** directed at a monogenetic cause of PD such as LRRK2, GBA, or parkin for treatment of Parkinson’s disease. | *Approvals must be announced at accessdata.fda.gov with an approval date on or before the specified date.  **The gene therapy must be non-alpha synuclein Parkinson-related, and use a viral vector or liposome to deliver the therapy (i.e. directed at a gene that can cause PD such as LRRK2, GBA, parkin, etc.). |
| Precision Medicine Therapy | Trial Enrolment; results | A rigorous phase 2 or phase 3 clinical trial* in Parkinson's disease that specifies eligibility based on GBA** mutational status successfully enrolls at least 80 subjects.*** | *Registered on clinicaltrials.gov with a “first posted” date on or before the date specified. The trial must be a randomized controlled trial with a sham/placebo arm and must have long-term follow up of at least 2 years using both clinical and imaging measures.  **GBA stands for Glucocerebrosidase, patients with Parkinson’s disease and associated Lewy body disorders have an increased frequency of GBA mutations compared to control individuals (Sidransky E, et al. The link between the GBA gene and parkinsonism. Lancet Neurol. 2012;11(11):986-998.).  ***As determined by a press release from the sponsor, a final enrollment record on clinicaltrials.gov, a published abstract or article available on PubMed, or direct confirmation from contacts listed on clinicaltrials.gov. |
| Cell Therapy | Trial initiation; results | The launch* of a rigorous phase 2 or phase 3 clinical trial** involving implantation of Parkinson's patients with dopaminergic cells derived from pluripotent stem cells.*** | *‘Launch’ means a trial is registered on clinicaltrials.govwith a “first posted” date on or before the date specified.  ** The trial must be a randomized controlled trial with a sham/placebo arm, must have long-term follow up of at least 2 years using both clinical and imaging measures, and must plan to enroll at least 120 patients.  ***A dopamine cell that has been made from a human embryonic stem cell or induced pluripotent stem cell source. |
| Imaging | Trial initiation; results | A selective alpha synuclein imaging agent* is integrated into a rigorous Parkinson's interventional clinical trial.** | *PET (positron emission topography) radiotracer to image the distribution of alpha-synuclein within the brain (https://www.michaeljfox.org/foundation/grant-detail.php?grant_id=845)  **The trial must be a randomized controlled trial with a sham/placebo arm, must have long-term follow up of at least 2 years using both clinical and imaging measures, must enroll at least 120 patients, and must be registered on clinicaltrials.gov with a “first posted” date on or before the date specified. |
| Deep Brain Stimulation | FDA approval | FDA approval* of the first closed-loop deep brain stimulation** device for the management of Parkinson’s disease. | *Approvals must be announced at accessdata.fda.gov with an approval date on or before the specified date.  **Open-loop devices stimulate at constant intensity regardless of current symptomology, while development of a closed-loop device will allow for an implanted neurostimulator that will use sensors and real-time modulation to determine the level of stimulation required based on patient symptoms at that moment in time (http://brl.ee.washington.edu/neural-engineering/closed-loop-dbs/). |
| Treatment for PD-MCI | Trial initiation; results | Launch* of a rigorous phase 3 clinical trial** testing a novel, non-cholinesterase inhibiting drug in the treatment of PD-MCI.*** | *Launch means a trial of the specified phase is registered on clinicaltrials.gov with a “first posted” date on or before the date specified.  ** The trial must be a randomized controlled trial with a sham/placebo arm, must have long-term follow up of at least 2 years using clinical measures, and must enroll at least 250 patients.  ***Mild cognitive impairment in Parkinson’s disease (PD-MCI) diagnostic criteria are defined based on the Movement Disorder Society (Litvan I, et al. Diagnostic Criteria for Mild Cognitive Impairment in Parkinson’s Disease: Movement Disorder Society Task Force Guidelines. Movement Disorders. 2012; 27(3):349-356). |
| Drug re-positioning | Trial results | A rigorous phase 3 clinical trial* utilizing a repositioned medication** and aimed at slowing the progression of Parkinson's symptoms reports*** a positive outcome on a primary efficacy endpoint.**** | * The trial must be a randomized controlled trial with a sham/placebo arm, must have long-term follow up of at least 2 years using both clinical and imaging measures, and must enroll at least 250 patients.  **A drug developed and FDA approved for one disorder and used to treat or manage a completely different disorder.  ***Results must be accessible on PubMed or clinicaltrials.gov with a date on or before the date specified.  ****As defined by statistical significance on all prespecified primary efficacy endpoint(s) that were prospectively registered on clinicaltrials.gov; at least one of the primary endpoints must use a common Parkinson's disease efficacy measure. |
| Exercise therapy | Trial results | A rigorous phase 2 or phase 3 clinical trial* testing the effect of exercises, physical activity, or physical therapy on Parkinson's progression reports** a positive outcome on a primary efficacy endpoint.*** | * The trial must be a randomized controlled trial with sham/placebo arm, must have long-term follow up of at least 2 years using both clinical and imaging measures, and must enroll at least 120 patients. **Results must be accessible on PubMed or clinicaltrials.govwith a date on or before the date specified.  ***As defined by statistical significance on all prespecified primary endpoint(s) that were prospectively registered on clinicaltrials.gov; at least one of the primary endpoints must use a common Parkinson's disease efficacy measure. |
| Body worn sensors | Clinical practice guideline recommendation | An algorithm derived from a body worn sensor* is accepted by the International Parkinson and Movement Disorder Society as a valid measure** of Parkinson's disease symptoms. | *Such as wrist sensors, shoe insoles and smartphone apps.  **Announced as an acceptable measure at either an MDS conference, in the MDS journal Movement Disorders, or in news and updates on www.movementdisorders.org. |
| Basic science discovery | Awarded *Science* magazine’s Breakthrough of the Year | *Science* magazine awards "Breakthrough of the Year"* to a molecule, process, cell, or discovery that is expressly described, in the accompanying Science article, as implicated in Parkinson's disease pathogenesis or possible treatment. | *For more information on Breakthrough of the Year: http://vis.sciencemag.org/breakthrough2017/. |
| Immuno-therapy | Trial results | A rigorous phase 2 or phase 3 clinical trial* testing an alpha-synuclein based immunotherapy** for Parkinson’s disease reports*** a positive outcome on a primary efficacy endpoint.**** | *The trial must be a randomized controlled trial with a sham/placebo arm, must have long-term follow up of at least 2 years using both clinical and imaging measures, and must enroll at least 120 patients.  **Immunotherapy could be active or passive. ***Results must be accessible on PubMed or clinicaltrials.gov with a date on or before the date specified.  **** As defined by statistical significance on all prespecified primary efficacy endpoint(s) that were prospectively registered on clinicaltrials.gov; at least one of the primary endpoints must use a common Parkinson's disease efficacy measure. |

**Table 2A**: List of milestones used in the expert survey including footnotes.

| **Milestone** | **Lay Mean (SD)** | **Expert Mean (SD)** | **P-value** |
| --- | --- | --- | --- |
| Monogenic Gene Therapy | 11.41 (15.66) | 7.29 (10.16) | <0.001* |
| Precision Medicine Therapy | 23.12 (23.54) | 23.99 (26.98) | 0.70 |
| Cell Therapy | 16.78 (19.61) | 15.62 (19.59) | 0.51 |
| Imaging | 21.77 (21.14) | 14.31 (16.63) | <0.001* |
| Deep Brain Stimulation | 20.46 (21.68) | 19.27 (22.32) | 0.55 |
| Treatment for PD-MCI | 19.18 (21.22) | 16.86 (19.13) | 0.20 |
| Drug repositioning | 20.83 (20.19) | 13.90 (15.90) | <0.001* |
| Exercise therapy | 39.06 (29.70) | 23.59 (22.88) | <0.001* |
| Body worn sensors | 29.62 (26.45) | 22.39 (23.65) | 0.001* |
| Basic science discovery | 16.26 (19.63) | 12.70 (14.77) | 0.02 |
| Immunotherapy | 16.83 (18.56) | 11.40 (14.62) | <0.001* |

**Table 3A:** Means and standard deviations for probability of the milestone occurring in the next 2 years for each milestone for laypeople and experts.

| **Milestone** | **Lay Mean (SD)** | **Expert Mean (SD)** | **P-value** |
| --- | --- | --- | --- |
| Monogenic Gene Therapy | 69.01 (25.78) | 58.07 (27.76) | <0.001* |
| Precision Medicine Therapy | 78.84 (22.88) | 80.16 (22.04) | 0.51 |
| Cell Therapy | 75.84 (22.93) | 69.09 (26.48) | 0.002* |
| Imaging | 80.99 (19.51) | 75.99 (24.55) | 0.01 |
| Deep Brain Stimulation | 79.45 (21.84) | 80.66 (22.16) | 0.54 |
| Treatment for PD-MCI | 78.92 (20.81) | 73.86 (23.83) | 0.01 |
| Drug repositioning | 78.59 (21.75) | 69.14 (26.00) | <0.001* |
| Exercise therapy | 86.27 (20.81) | 78.48 (26.47) | <0.001* |
| Body worn sensors | 85.06 (19.85) | 83.92 (22.27) | 0.54 |
| Basic science discovery | 72.13 (25.04) | 65.26 (28.33) | 0.004* |
| Immunotherapy | 75.29 (23.18) | 62.53 (28.15) | <0.001* |

**Table 4A:** Means and standard deviations for probability of the milestone occurring in the next 10 years for each milestone for laypeople and experts.

| **Milestone** | **Aggregated Expert Forecast** | **Average Difference for Lay Opinion (Confidence Interval)** | **P-value** |
| --- | --- | --- | --- |
| Gene Therapy | 26.27 | 6.88 (4.40, 9.42) | <0.001* |
| Precision Medicine Therapy | 53.75 | -4.14 (-6.95, -1.31) | 0.004* |
| Cell Therapy | 38.90 | 4.94 (2.19, 7.73) | <0.001* |
| Imaging | 43.39 | 6.67 (4.03, 9.30) | <0.001* |
| Deep Brain Stimulation | 50.66 | -1.86 (-4.57, 0.87) | 0.17 |
| Treatment for PD-MCI | 44.97 | 1.10 (-1.47, 3.71) | 0.40 |
| Drug repositioning | 38.79 | 9.63 (6.93, 12.34) | <0.001* |
| Exercise therapy | 52.58 | 10.92 (8.07, 13.74) | <0.001* |
| Body worn sensors | 56.87 | 0.25 (-2.53, 3.03) | 0.86 |
| Basic science discovery | 36.47 | 4.01 (1.26, 6.79) | 0.005 |
| Immunotherapy | 35.45 | 8.72 (6.22, 11.25) | <0.001* |

**Table 5A**: Difference between the average lay forecast and the aggregated expert forecasts for each milestone along with a 95% confidence interval. Positive values indicate optimism relative to experts. P-values are noted with an asterisk when they were statistically significant in a two-sided test at the 0.05 level with Bonferroni correction for the individual 11 milestones. The last four rows correspond to the questions about trial results.

References

1. Karvetski CW, Olson KC, Mandel DR, Twardy CR. Probabilistic Coherence Weighting for Optimizing Expert Forecasts. *Decision Analysis*. 2013;10(4):305-326. doi:10.1287/deca.2013.0279

2. Fan Y, Budescu DV, Mandel D, Himmelstein M. Improving Accuracy by Coherence Weighting of Direct and Ratio Probability Judgments. *Decision Analysis*. 2019;16(3):197-217. doi:10.1287/deca.2018.0388

3. Kane PB, Benjamin DM, Barker RA, Lang AE, Sherer T, Kimmelman J. Forecasts for the Attainment of Major Research Milestones in Parkinson’s Disease. *J Parkinsons Dis*. Published online April 24, 2020. doi:10.3233/JPD-201933
